# The Cost of Inequity: Financial Burden of Neighborhood Disparities in Pediatric Critical Care Use

**DOI:** 10.1016/j.chstcc.2026.100251

**Published:** 2026-05-07

**Authors:** Carlie N. Myers, Qing Duan, Carley L. Riley, Andrew F. Beck

**Affiliations:** Department of Pediatrics (C. N. M., Q. D., C. R., and A. F. B.), University of Cincinnati College of Medicine, the Division of Critical Care (C. N. M. and C. R.), the Division of Biostatistics and Epidemiology (Q. D.), the Office of Population Health (A. F. B.), the Michael Fisher Child Health Equity Center (C. R. and A. F. B.), the Division of General and Community Pediatrics (A. F. B.), and the Division of Hospital Medicine (A. F. B.), Cincinnati Children’s Hospital Medical Center, Cincinnati, OH.

**Keywords:** cost, disparities, neighborhood, PICU, utilization

## Abstract

**BACKGROUND:**

Disparities in health care access and use among critically ill pediatric populations disproportionately affect children from marginalized communities, leading to worse health outcomes and increased financial burden. Neighborhood-level factors likely contribute to variations in PICU admission rates, length of stay (LOS), and costs. However, the impact of such factors on PICU costs at the population-level remains understudied.

**RESEARCH QUESTION:**

What variations in PICU use (admission rates, prolonged LOS, and PICU costs) exist in low-opportunity neighborhoods compared with high-opportunity neighborhoods?

**STUDY DESIGN AND METHODS:**

This population-level retrospective cohort study enrolled patients younger than 18 years admitted to our PICU from January 1, 2016, through December 31, 2022, with residential addresses in the primary service area (specific counties in Ohio, Kentucky, or Indiana). Patient addresses were linked to census tract-level Child Opportunity Index (COI) data. Associations between COI quintiles and PICU use, including admission rates, LOS, PICU costs, and diagnostic patterns, were examined. Bivariate associations were tested using the Wilcoxon rank-sum test. Linear regression models were used to estimate associations between COI and PICU use outcomes, adjusting for chronic complex conditions.

**RESULTS:**

Nine thousand six hundred thirty-four PICU encounters occurred among children residing in 491 regional census tracts. Very low-opportunity tracts showed higher PICU admission rates (3.41 per 1,000 children vs 1.76 per 1,000 children) and greater annual PICU cost per child ($92 vs $60) compared with peers in very high-opportunity tracts (*P* < .001); but no differences in LOS were found. Respiratory diagnoses were the most common reason for admission, with disproportionately higher rates in low-opportunity tracts (39% vs 31%; *P* < .001). Other diagnostic categories, including neurologic, trauma, genetic, and endocrine, varied across COI levels.

**INTERPRETATION:**

Our findings suggest that structural inequities and neighborhood conditions contribute to PICU use and potentially avoidable health care costs. Addressing upstream social determinants of health through neighborhood-based investments may reduce PICU use and may advance child health.

Disparities in health care access and use are well described within critically ill pediatric populations.^1–3^ Inequities in health care delivery and resource allocation shape a range of patient outcomes, including disparities in morbidity and mortality^4,5^ that disproportionately affect pediatric patients from marginalized and socioeconomically disadvantaged communities. This can lead to preventable morbidity, mortality, and financial strain on families and health care systems. Beyond the clinical effects, disparities in PICU use contribute to significant direct and indirect health care costs borne by those with interests across the health care system,^6,7^ compounding broader issues of injustice and inequity. PICU care is among the most resource-intensive components of pediatric health care delivery,^8,9^ with high per-patient costs driven by specialized staffing, advanced medical technologies, and prolonged hospital lengths of stay (LOSs). As a result, variations in PICU admission rates and LOSs across different populations have substantial financial implications, affecting not only families, but also health care systems, insurers, government payers, and society at large.^6^

Addressing disparities in PICU use presents a critical opportunity for targeted interventions to leverage community resources and partnerships to improve pediatric health.^10^ Community health advocates, health care institutions, and policymakers increasingly are focused on neighborhood-based strategies to optimize pediatric health care delivery while mitigating unnecessary, potentially preventable health care costs. Yet, a gap remains in understanding how neighborhood-level factors contribute to variations in PICU use. Social determinants of health, including access to primary and preventive care, healthy food, safe housing, environmental exposures, socioeconomic stability, and neighborhood-level health care resources^1–5,11,12^, likely influence incidence of critical illnesses and the need for PICU admission. Children from lower opportunity neighborhoods, which often are characterized by higher rates of poverty, limited health care infrastructure, and greater psychosocial stressors, may be at increased risk for delayed care and exacerbation of preventable conditions.^11,13–15^ Consequently, these children may experience longer PICU stays, increased medical complexity, and higher associated PICU costs, further reinforcing cycles of health and economic disadvantage.

With growing interest in the intersection of such determinants and pediatric critical care, studies have systematically examined the relationship between neighborhood-level opportunity and PICU use; however, to our knowledge, none specifically have linked neighborhood opportunity with variability in PICU outcomes measured at the population level. This knowledge gap limits the ability of health care systems to develop data-driven interventions that reduce disparities and increase equity in resource allocation. To fill this gap, our study tested the hypothesis that low opportunity neighborhoods experience disparities in PICU use, including higher admission rates, prolonged LOS, and increased PICU costs. By explicitly linking neighborhood opportunity with PICU costs, our study adds a novel dimension to the existing literature and underscores the financial and equity implications of neighborhood-level disparities in pediatric critical care. Findings may inform health care delivery and policy reforms aimed at reducing health care disparities, eliminating potentially avoidable expenditures, and advancing health justice through cost-effective, community-informed models of care.

## Study Design and Methods

### Study Design, Setting, and Population

We conducted a population-level retrospective observational study of pediatric patients admitted to the Cincinnati Children’s Hospital Medical Center PICU, a large quaternary-care, free-standing children’s hospital in southwestern Ohio. The Cincinnati Children’s institutional review board approved this study on April 4, 2022, with a waiver of informed consent (Identifier: MOD00031986). All procedures adhered to institutional ethical standards and the tenets of the Helsinki Declaration of 1975.

Patients < 18 years of age admitted to our PICU from January 1, 2016, through December 31, 2022, with a residential address in our 14-county primary service area on the day of the PICU admission were included. The primary service area includes counties in Ohio (Adams, Brown, Butler, Clermont, Clinton, Hamilton, Highland, and Warren), Kentucky (Boone, Campbell, and Kenton), and Indiana (Dearborn, Franklin, and Ripley). The PICU at our institution is the only site for pediatric critical care provision within the primary service area. We excluded patients who were houseless or wards of the state. To reduce bias from extreme outliers, PICU admissions of > 365 days (n = 34) were removed from analysis because they additionally may reflect individual and institutional-level factors. Census tracts with fewer than 3 PICU admissions during the study period were excluded to avoid unstable small-area estimates for admission rates,^16^ LOS, and median PICU cost; < 3% of census tracts within the primary service area were excluded. All admissions, including readmissions, were included in the analysis. Patient-level demographic, clinical, and financial claims data were extracted from the electronic health record. Addresses linked to each encounter at the time of admission were geocoded and spatially joined to corresponding census tracts. We then appended tract-level Child Opportunity Index (COI) 3.0 data.^17^ The COI is a validated multidimensional tool used to characterize areas like census tracts for overall opportunity, including modifiable risk factors across specific domains: education, health and environment, and social and economic. Hospital-level health care costs were estimated from charges using the year (2016–2022) and hospital-specific cost-to-charge ratios from the Centers for Medicare and Medicaid Services cost reports in accordance with the Healthcare Cost and Utilization Project Cost-to-Charge Ratio Methodologies.^18^

### Measures

Primary outcomes included markers of PICU use captured at the census tract level: LOS, PICU admission rate, PICU cost per census tract child, and median PICU cost per census tract. LOS was defined as the number of days of PICU admission. Admission rates were calculated as PICU admissions per 1,000 children (< 18 years of age) per census tract. PICU cost per census tract child was calculated by dividing total PICU expenditures within a census tract by the population of youth < 18 years of age. PICU costs per census tract child were averaged annually over the study period. The median PICU cost reflected the median cost per PICU encounter within each census tract. The primary exposure was the COI, categorized into state-normed quintiles (very high, high, moderate, low, and very low). State-normed quintiles were derived by ranking all census tracts within the state (Ohio, Kentucky, or Indiana) and assigning quintiles, such that a census tract’s COI reflected its opportunity relative to all the other census tracts statewide.^17^ Patient-level variables, used to describe PICU encounter demographics; included age; insurance type; International Classification of Diseases, 10th Revision, diagnosis codes; presence of pediatric complex chronic conditions (CCCs)^19–23^; and illness severity (Pediatric Risk of Mortality III scores). We extracted primary International Classification of Diseases, 10th Revision, diagnosis codes from the electronic health record and applied the PCCC R package (version 1.0.7)^24^ to calculate the total number of CCCs per encounter^25^; total CCCs were treated as a covariate in the analysis. Race and ethnicity, treated as social constructs, were self-reported or parent-reported and were recorded in the electronic health record by administrative staff.

### Statistical Analysis

We summarized patient-level demographics and clinical characteristics for all PICU encounters overall and by insurance type. The primary outcomes were summarized and compared across state-normed COI quintiles. Descriptive statistics were presented as medians and interquartile ranges for continuous variables and as frequencies and percentages for categorical variables. Bivariate associations between patient characteristics and insurance type were assessed using the Wilcoxon rank-sum test for continuous variables and the Pearson χ^2^ test for categorical variables. The Kruskal-Wallis rank-sum test was used to compare outcomes across COI quintiles. Associations between PICU admission rate and COI, adjusted for medical complexity (presence of > 0 CCCs and > 1 CCCs), were estimated using a negative binomial model with an offset for census tract level population of children < 18 years of age (per 1,000 children). For use outcomes, linear regression models were used to estimate associations between COI and log-transformed PICU use outcomes while accounting for CCCs. Analyses of census tract-level outcomes were limited to bivariate comparisons, consistent with our ecological, population-level analytic approach. Multivariable regression was not performed for census tract-level outcomes because the census tract was the primary unit of analysis and the number of tracts limited the inclusion of multiple covariates without risk of overfitting. All analyses were conducted using R version 4.2.2 software (R Foundation for Statistical Computing).

## Results

We identified 9,634 unique PICU admissions experienced by patients living across 491 regional census tracts during the study period. The median age at the time of admission was 59 months (interquartile range, 14–157 months), 4,234 patients (45%) who contributed encounters were female, 6,324 patients (66%) identified as White, and 5,464 patients (58%) were publicly insured (Table 1). In the cohort, 58% had ≥ 1 CCC. PICU diagnosis category distributions varied significantly across neighborhood opportunity quartiles. Respiratory diagnoses were the most common reason for PICU admissions (a median of 33% of all PICU encounters per census tract). The relative proportion of respiratory diagnoses were higher in very low opportunity census tracts (39%) compared with very high opportunity census tracts (31%; *P* < .001) (Table 2). Neurologic, trauma, genetic, endocrine, mental health, and oncologic diagnoses also showed significant variation by neighborhood opportunity (*P* < .05 for all). In contrast, infectious diagnoses did not differ significantly across census tract COI quintiles (*P* = .451) (Table 2). Patient-level Pediatric Risk of Mortality III scores were higher among children in lower opportunity neighborhoods. The Pediatric Risk of Mortality III scores in this cohort are comparable with those of reference PICUs.

**Figure 1 –:**
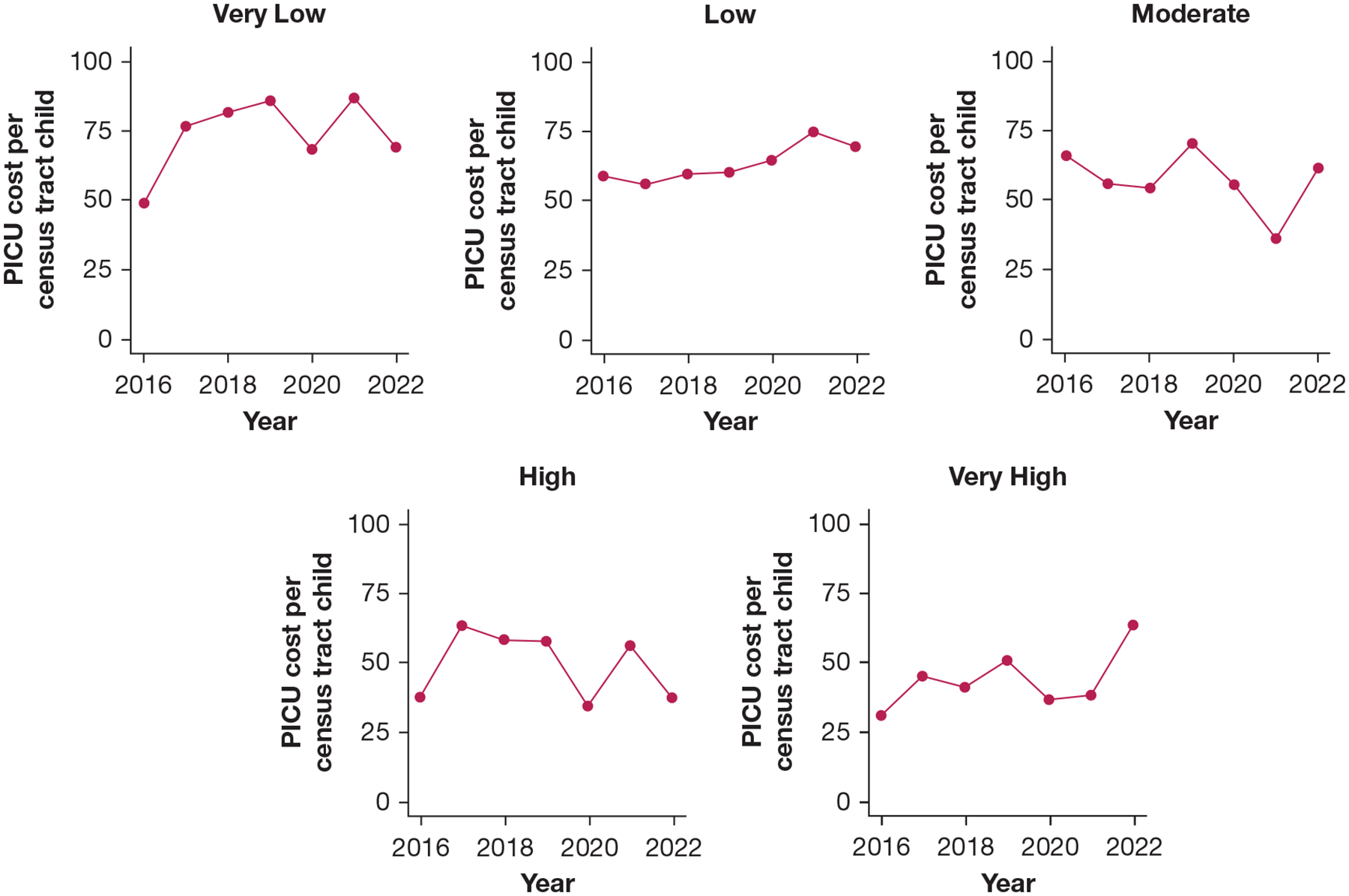
A-E, Graphs showing yearly PICU cost per census tract child according to Child Opportunity Index quintile: Very low (A), Low (B), Moderate (C), High (D), and Very high (E).

Both PICU admission rates and yearly PICU cost per census tract child decreased significantly with increasing neighborhood opportunity (*P* < .001) (Table 3), even when adjusting for total CCCs (Table 4). Children from very low opportunity neighborhoods showed the highest admission rates (3.41 per 1,000 children) (Table 3) and highest yearly PICU cost per census tract child ($92) (Fig 1) compared with those from very high opportunity neighborhoods (1.76 per 1,000 children and $60, respectively) (Table 3, Fig 1). No significant differences were found across opportunity levels in median PICU cost per census tract or LOS (Table 2) or in adjusted models for total CCCs (Table 4).

## Discussion

Our findings suggest that disparities in PICU use are associated with neighborhood-level child opportunity, with children in very low opportunity neighborhoods experiencing higher admission rates and greater per-neighborhood costs than those from high opportunity neighborhoods. These differences occurred despite heterogeneity in the population and no significant variation in LOS or median per-encounter costs. Taken together, this pattern may indicate that increased aggregate costs in lower opportunity neighborhoods are driven primarily by more frequent admissions, rather than greater resource use per encounter. Although we cannot infer causality from these results, they suggest that structural and social determinants of health likely contribute to higher PICU use.

These observations noted in our analysis add to the existing literature on inequities in pediatric health and critical care. Communities characterized by lower neighborhood opportunity often have reduced access to preventive and primary care, higher rates of environmental exposures, and increased psychosocial stressors.^12^ Such factors may contribute to a greater burden of preventable illness requiring PICU admission.^26,27^ Although our study did not explore causal mechanisms, the higher PICU use observed in disadvantaged neighborhoods highlights potential areas for upstream public health and health care system investment.

Respiratory and endocrine diagnoses were the most common categories of PICU encounters and occurred more frequently among children from very low opportunity neighborhoods. This finding is consistent with prior research linking respiratory morbidity, including asthma, to environmental conditions such as housing quality and air quality, as well as gaps in outpatient management. Targeted interventions, such as school-based asthma programs,^28^ telemedicine, and environmental health initiatives, have demonstrated benefit in other settings and could be evaluated as potential strategies to reduce disparities in PICU use.

The absence of differences in LOS or per-encounter costs across neighborhood opportunity levels may reflect standardized care often administered in the PICU as soon as children are admitted. However, the higher admission rates observed in low opportunity neighborhoods suggest that increased use may reflect conditions that could potentially be managed earlier in outpatient settings or prevented via upstream intervention. Further study is needed to better understand the relationship between such factors and downstream intensive care use.

### Sociomedical Context of Pediatric Critical Illness

Neighborhood conditions are increasingly recognized as important determinants of pediatric health and critical illness.^12,29^ By linking PICU use data with the COI, our study provides population-level evidence that neighborhood-level opportunity is associated with variation in intensive care use and costs. Although we cannot account for all confounders, these findings reinforce the importance of considering place and social context in efforts to improve pediatric health equity.^30,31^

### Disparity Reduction Interventions and Community-Based Investment

Hospitals and health care systems have a role to play in addressing both social and medical needs in ways that promote optimal, equitable outcomes for both individuals and communities. Modifications to the health care system, including practices to tailor services for those most at risk, may alleviate the negative impact of social determinants of health and the financial strain of PICU use. Interventions to reduce PICU admission rate disparities would benefit from a co-designed approach pursued in conjunction with frontline PICU staff and community-based interest holders, then applied at scale. The literature suggests that novel changes to health care systems, such as pediatric-focused care coordination and medical-community partnerships,^10^ can proactively address unmet social needs like housing insecurity, food access, and insurance instability, which often are associated with critical illness.^12,32^ Patient-centered models, including medical-legal partnerships, family navigation services, and neighborhood-based case management, have shown particular promise.^33^ In parallel, collaborative efforts between health care systems and neighborhood-based supports have leveraged home visits, environmental remediation education, and school-based outreach. Together, these approaches may translate into lower PICU admissions in low-opportunity neighborhoods. Telemedicine programs integrated with pediatric primary care practices have improved access to timely care and medication adherence for children with chronic conditions such as asthma and diabetes, conditions frequently contributing to PICU admissions in low-opportunity neighborhoods.

Such programs can be enhanced through home monitoring technologies, particularly in neighborhoods with limited geographic access to subspecialty care, potentially reducing disparities and immediate cost savings for high-risk, high-cost pediatric patients.^34,35^ More robust adaptations to transition-of-care protocols, including home health services for low-opportunity and rural neighborhoods, post-PICU check-ins, and community health worker outreach, also have demonstrated promise, with some studies showing reduced hospital readmissions when short-term, nurse-led pediatric care is delivered in homes after discharge.^36^

Disparity reduction initiatives must prioritize capital investments further in historically marginalized neighborhoods. Currently, many investments remain reactive and focused on managing downstream consequences of structural inequities, such as costly PICU admissions, rather than reallocating resources upstream to reduce the estimated yearly $92 per census tract child spent on intensive care services.

Evidence demonstrates that investments that reduce poverty and direct resources toward young children yield particularly high long-term returns, with some programs generating up to $10 in community benefits for every dollar invested.^37,38^ Economic payoffs seem greatest for interventions addressing child health, early care and education, and K through 12 education, which have been shown to improve population health outcomes and to increase future tax revenue by more than their upfront costs.^38–41^

Strategic neighborhood-level investment in affordable housing, lending opportunities, small business development, and job creation can address the social determinants of health that contribute to high PICU use. Health care systems are well positioned to support such upstream resource allocation using community benefit dollars to target institutional racism and entrenched poverty, each critical root causes of poor child health outcomes.^42,43^ Although neighborhood-level poverty, housing quality, the built environment, and vehicle access all have been associated with increased PICU use, neighborhood-level interventions to reduce disparities have yet to be described; further examination of markers of neighborhood resilience may lead to sustainable, community-based interventions, and future studies should design and test such interventions in collaboration with community members to reduce disparities in PICU use.

### Study Limitations

Our study has 5 main limitations. First, this is a cross-sectional study with results that are associational and not causal. Second, this is an ecological population-level analysis without application to patient-level encounters. Third, although the COI is a validated tool, it cannot capture the full heterogeneity within census tracts or account for individual-level adversity. Fourth, our analysis is limited to a single region and health care system, which may affect generalizability, although our findings likely mirror trends in other urban and semiurban areas.^44^ Finally, our cost estimates are derived from administrative cost-to-charge ratios, which are hospital-level costs and may not fully reflect true societal costs or downstream impacts on families.

## Interpretation

Our findings support neighborhood-based health investment, preventive care, and cross-sector collaboration to mitigate avoidable pediatric critical illness and its financial consequences. Identifying and addressing the root causes of pediatric critical care disparities can help health care systems and policymakers to prioritize value-based, equitable care.

## Figures and Tables

**TABLE 1 ]:** Patient Demographic and Clinical Data for All PICU Encounters

Patient Characteristics	All Encounters (n = 9,634)	Public Insurance (n = 5,464)	Other Insurance (n = 4,170)	*P* Value
Age, mo	59 (14–157)	46 (12–142)	83 (19–172)	< .001^a^
Sex				.037^b^
Female	4,324 (45)	2,402 (44)	1,922 (46)	
Male	5,310 (55)	3,062 (56)	2,248 (54)	
Race				< .001^b^
White	6,324 (66)	2,899 (53)	3,425 (82)	
Black	2,269 (24)	1,898 (35)	371 (8.9)	
Multiracial	354 (3.7)	244 (4.5)	110 (2.6)	
Asian	170 (1.8)	69 (1.3)	101 (2.4)	
Other	291 (3.0)	197 (3.6)	94 (2.3)	
Missing or unknown	226 (2.3)	157 (2.9)	69 (1.7)	
Ethnicity				< .001^b^
Non-Hispanic	9,001 (93)	4,995 (91)	4,006 (96)	
Hispanic	571 (5.9)	437 (8.0)	134 (3.2%)	
Missing or unknown	62 (0.6)	32 (0.6)	30 (0.7)	
Primary language				< .001^b^
English	8,986 (93)	5,083 (93)	3,903 (94)	
Spanish	298 (3)	255 (4.7)	34 (0.8)	
Arabic	205 (2.1)	27 (0.5)	178 (4.3)	
Sign language	29 (0.3)	10 (0.2)	19 (0.5)	
Other	125 (1.3)	89 (1.6)	36 (0.9)	
Missing or unknown	0	0	0	
Total No. of CCCs				< .001^b^
0	4,091 (42)	2,408 (44)	1,683 (40)	
1	2,279 (24)	1,249 (23)	1,030 (25)	
2–3	1,946 (20)	1,050 (19)	896 (21)	
≥ 3	1,318 (14)	757 (14)	561 (13)	

Data are presented as No. (%) or median (interquartile range) unless otherwise indicated. CCC = complex chronic condition.

a Wilcoxon rank-sum test.

b Pearson χ^2^ test.

**TABLE 2 ]:** Fraction of PICU Encounter Diagnoses Stratified by COI Quintiles

Diagnosis Category	Overall	State-Normed COI Category					
		Very Low (n = 131)	Low (n = 99)	Moderate (n = 68)	High (n = 92)	Very High (n = 101)	*P* Value^a^
Respiratory	0.33 (0.25–0.44)	0.39 (0.29–0.50)	0.33 (0.22–0.42)	0.32 (0.22–0.47)	0.30 (0.21–0.38)	0.31 (0.22–0.40)	**< .001**
Neurologic	0.10 (0.04–0.15)	0.09 (0.03–0.13)	0.10 (0.03–0.18)	0.07 (0.00–0.12)	0.11 (0.08–0.17)	0.10 (0.05–0.15)	**.010**
Trauma	0.09 (0.04–0.15)	0.09 (0.00–0.14)	0.11 (0.04–0.18)	0.10 (0.03–0.17)	0.10 (0.06–0.15)	0.07 (0.00–0.13)	**.013**
Genetic	0.05 (0.00–0.10)	0.03 (0.00–0.06)	0.05 (0.00–0.11)	0.04 (0.00–0.11)	0.06 (0.00–0.11)	0.06 (0.00–0.11)	**.010**
Infectious	0.04 (0.00–0.08)	0.03 (0.00–0.08)	0.04 (0.00–0.07)	0.04 (0.00–0.07)	0.05 (0.00–0.10)	0.05 (0.00–0.10)	.451
Endocrine	0.04 (0.00–0.09)	0.04 (0.00–0.11)	0.05 (0.00–0.11)	0.06 (0.00–0.10)	0.03 (0.00–0.08)	0.02 (0.00–0.07)	**.024**
Mental health	0.03 (0.00–0.08)	0.00 (0.00–0.07)	0.00 (0.00–0.06)	0.03 (0.00–0.08)	0.05 (0.00–0.08)	0.04 (0.00–0.10)	**.006**
Oncologic	0.00 (0.00–0.07)	0.00 (0.00–0.05)	0.00 (0.00–0.06)	0.00 (0.00–0.07)	0.03 (0.00–0.08)	0.04 (0.00–0.10)	**.028**

Data are presented as median (interquartile range) unless otherwise indicated. COI = Child Opportunity Index.

a Kruskal-Wallis rank-sum test.

**TABLE 3 ]:** PICU Admission Rates, LOS, and Cost Stratified by COI Quintiles

Characteristic	Overall (N = 490)	State-Normed COI Category					
		Very Low (n = 131)	Low (n = 99)	Moderate (n = 68)	High (n = 92)	Very High (n = 101)	*P* Value^a^
Admission rate, per 1,000 children	2.41 (1.66–3.45)	3.41 (2.26–4.91)	2.79 (2.08–3.45)	2.58 (1.83–3.44)	2.04 (1.50–2.59)	1.76 (1.42–2.41)	**< .001**
PICU cost per census tract child, $	76 (39–122)	92 (50–152)	80 (47–118)	84 (44–139)	61 (36–100)	60 (33–98)	**< .001**
Median PICU cost per encounter, $	17,724 (13,765–23,745)	16,541 (13,403–22,206)	17,982 (14,298–24,421)	17,436 (13,442–27,335)	18,208 (13,224–24,181)	18,208 (14,323–25,620)	.582
Median PICU LOS, d	4.11 (3.35–5.28)	3.99 (3.34–5.33)	3.87 (3.38–4.92)	4.24 (3.26–5.35)	4.13 (3.25–5.28)	4.24 (3.57–5.46)	.559
Severity of illness, PRISM 3 score	1.00 (0.00–3.00)	2.00 (0.00–3.00)	2.00 (0.00–3.00)	0.00 (0.00–2.00)	0.50 (0.00–3.00)	0.00 (0.00–2.00)	**.006**
Fraction of encounters with ≥ 1 CCCs	0.56 (0.44–0.67)	0.53 (0.40–0.63)	0.55 (0.46–0.63)	0.56 (0.44–0.69)	0.60 (0.45–0.71)	0.60 (0.46–0.71)	**.028**

Data are presented as median (interquartile range) unless otherwise indicated. CCC = complex chronic condition; COI = Child Opportunity Index; LOS = length of stay; PRISM = Pediatric Risk of Mortality.

a Kruskal-Wallis rank-sum test.

**TABLE 4 ]:** Multivariable Regression Models for PICU Use Outcomes Including Joint Tests

Outcome	Adjuster Model > 0 CCC					Adjusted Model > 1 CCC				
	Variable	IRR	95% CI	*P* Value	Joint Test *P* Value^a^	Variable	IRR	95% CI	*P* Value	Joint Test *P* Value^a^
Admission rate, per 1,000 children	State-normed COI				**< .001**	State-normed COI				**< .001**
	Very high (reference)	Ref.	Ref.			Very high (reference)	Ref.	Ref.		
	High	1.19	1.05–1.36	**.008**		High	1.18	1.04–1.35	.012	
	Moderate	1.50	1.30–1.73	**< .001**		Moderate	1.49	1.29–1.73	**< .001**	
	Low	1.54	1.35–1.75	**< .001**		Low	1.52	1.33–1.73	**< .001**	
	Very low	2.03	1.79–2.29	**< .001**		Very low	2.04	1.81–2.31	**< .001**	
	Proportion ot patients with > 0 CCC	1.77	1.38–2.26	**< .001**	**< .001**	Proportion ot patients with > 1 CCC	1.85	1.46–2.36	**< .001**	**< .001**
		Beta	95% CI	*P* value	Joint test *P* value		Beta	95% CI	*P*value	Joint test *P* value
PICU cost pe r census tract child, $	State-normed COI				**< .001**	State-normed COI				**< .001**
	Very high (reference)	Ref.	Ref.			Very high (reference)	Ref.	Ref.		
	High	0.05	−0.17 to 0.27	.654		High	0.03	−0.19 to 0.26	.769	
	Moderate	0.32	0.08–0.56	**.009**		Moderate	0.32	0.08–0.57	**.009**	
	Low	0.34	0.12–0.56	**.002**		Low	0.31	0.09–0.53	**.006**	
	Very low	0.55	0.34–0.75	**< .001**		Very low	0.56	0.35–0.76	**< .001**	
	Proportion ot patients with > 0 CCC	1.70	1.3–2.0	**< .001**		Proportion ot patients with > 1 CCC	1.60	1.2–2.0	**< .001**	**< .001**
		Beta	95% CI	*P* value	Joint test *P* value		Beta	95% CI	*P* value	Joint test *P* value
Median PICU cost per encounter, $	State-normed COI				.670	State-normed COI				.679
	Very high (reference)	Ref.	Ref.			Very high (reference)	Ref.	Ref.		
	High	−0.05	−0.17 to 0.07	.420		High	−0.06	−0.18 to 0.06	.341	
	Moderate	−0.01	−0.14 to 0.11	.830		Moderate	−0.01	−0.14 to 0.12	.833	
	Low	−0.02	−0.14 to 0.09	.674		Low	−0.05	−0.16 to 0.07	.445	
	Very low	−0.03	−0.14 to 0.08	.603		Very low	−0.03	−0.14 to 0.08	.645	
	Proportion ot patients with > 0 CCC	0.93	0.74–1.1	**< .001**	**< .001**	Proportion ot patients with > 1 CCC	0.89	0.69–1.1	**< .001**	**< .001**

CCC = complex chronic condition; COI = Child Opportunity Index; Ref. = reference.

a Multivariable regression models for PICU use outcomes including joint test *P* values.

## Take-Home Points

**Study Question:** Is Child Opportunity Index associated with differences in PICU admission rates, length of stay (LOS), and population-level PICU costs?

**Results:** Children living in very low-opportunity neighborhoods experienced higher PICU admission rates and greater PICU cost per census tract child compared with those in very high-opportunity neighborhoods, driven largely by respiratory diagnoses, with no differences in LOS or median per-encounter cost.

**Interpretation:** These findings suggest that neighborhood-level structural disadvantage contributes to higher downstream PICU use and disproportionate population-level spending, highlighting the potential for upstream, place-based investments to reduce avoidable critical illness and advance child health equity.

## ABBREVIATIONS:

CCC: complex chronic condition
COI: Child Opportunity Index
LOS: length of stay
